# Supplementary material for: Spatial distribution, and predictors of late initiation of first antenatal care visit in Ethiopia: Spatial and multilevel analysis
Source: PLoS One. 2023 Jul 25;18(7):e0288869. doi: 10.1371/journal.pone.0288869 (PMC10368282; doi:10.1371/journal.pone.0288869)
Supplement: Attachment — Submitted filename: Reviewers comments.docx [file pone.0288869.s001.docx]

Spatial distribution, and multilevel analysis of the predictors of late initiation of first antenatal care visit among reproductive-age women in Ethiopia: Using the Ethiopia Mini Demographic and Health Survey 2019

My concern

The idea of the work is sounding great. But I have the following minor comments

- Modify the topic
- The abstract is too long and gives a brief recommendation relying on your key findings
- Remove outdated references and support them with more updated references
- Try to match your objectives, methods, results, and conclusions

Decision: After making all the necessary updates accept the article for publications
